# The link between early life stress and psycho-cardio-metabolic multi-morbidity: Findings from The EarlyCause Consortium

**DOI:** 10.1192/j.eurpsy.2024.50

**Published:** 2024-08-27

**Authors:** C. Cecil

**Affiliations:** Child and Adolescent Psychiatry, Erasmus MC, Rotterdam, Netherlands

## Abstract

**Abstract:**

In this talk I will present new findings from EarlyCause, a European consortium which aims to better understand the link between early life stress and the development of psycho-cardiometabolic (PCM) comorbidity across the lifespan, leveraging data from large-scale pediatric and adult population studies. I will discuss findings regarding the effect of (prenatal and postnatal) early life stress on PCM health outcomes and their comorbidity, potential moderating and mediating factors, as well as evidence for causality.

**Disclosure of Interest:**

None Declared

